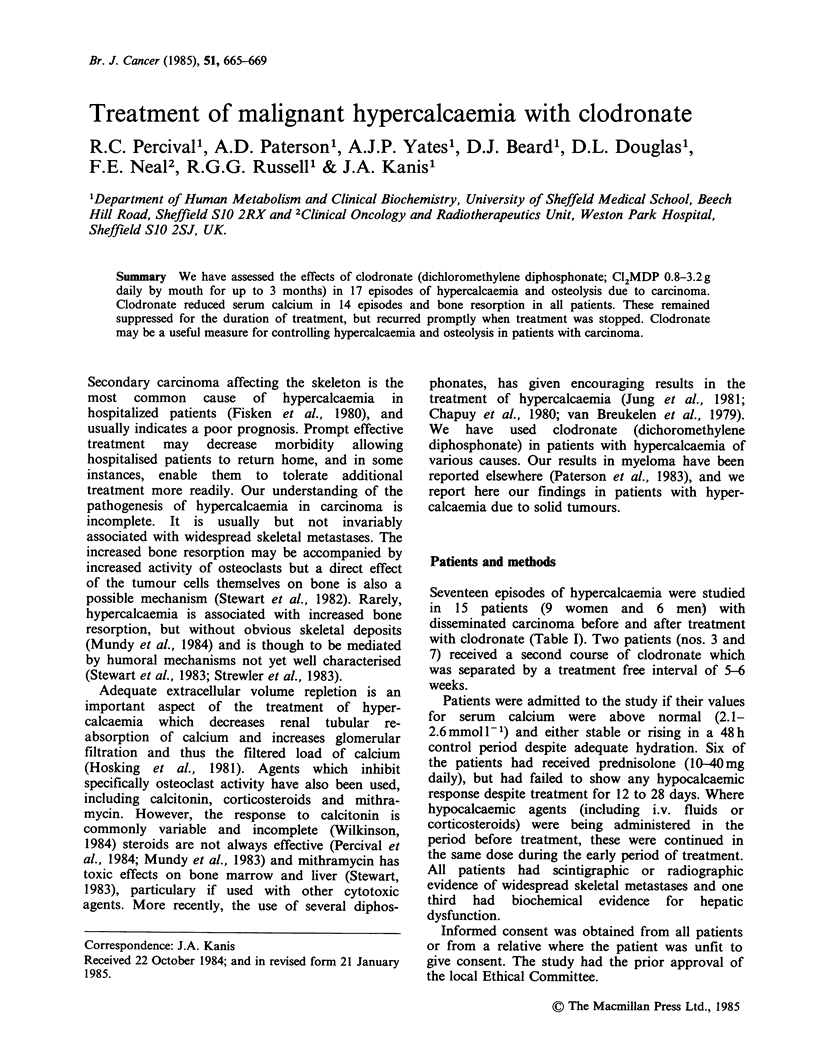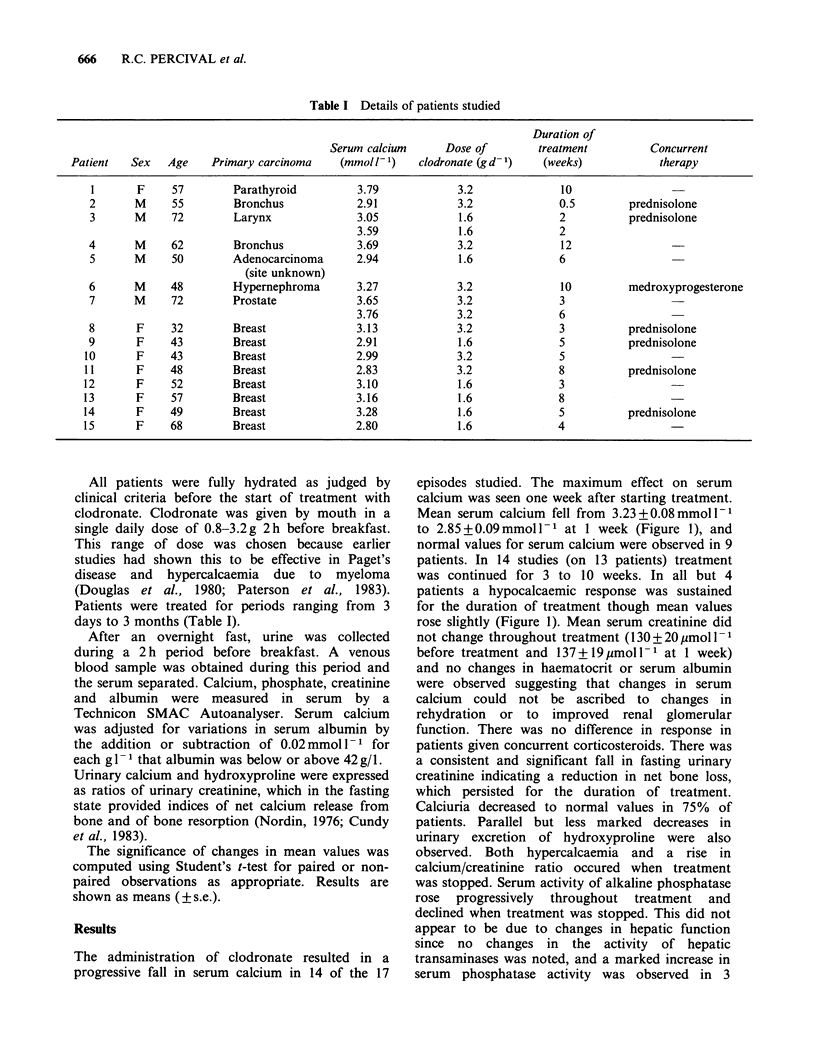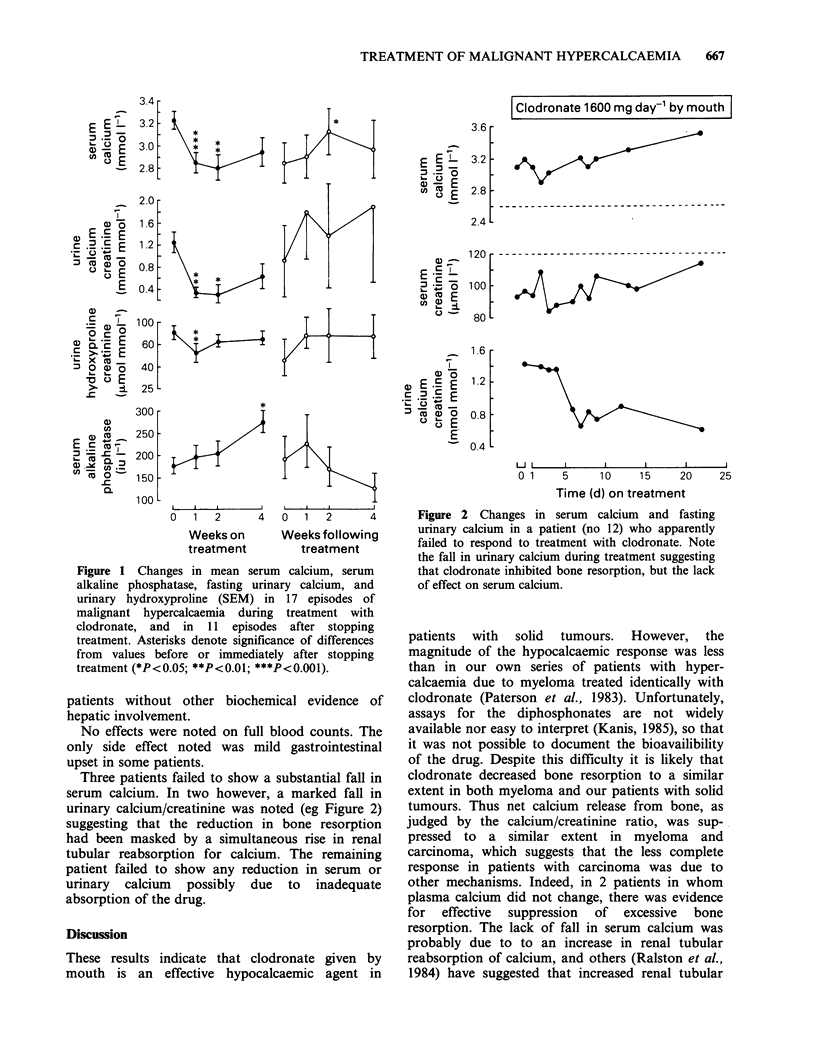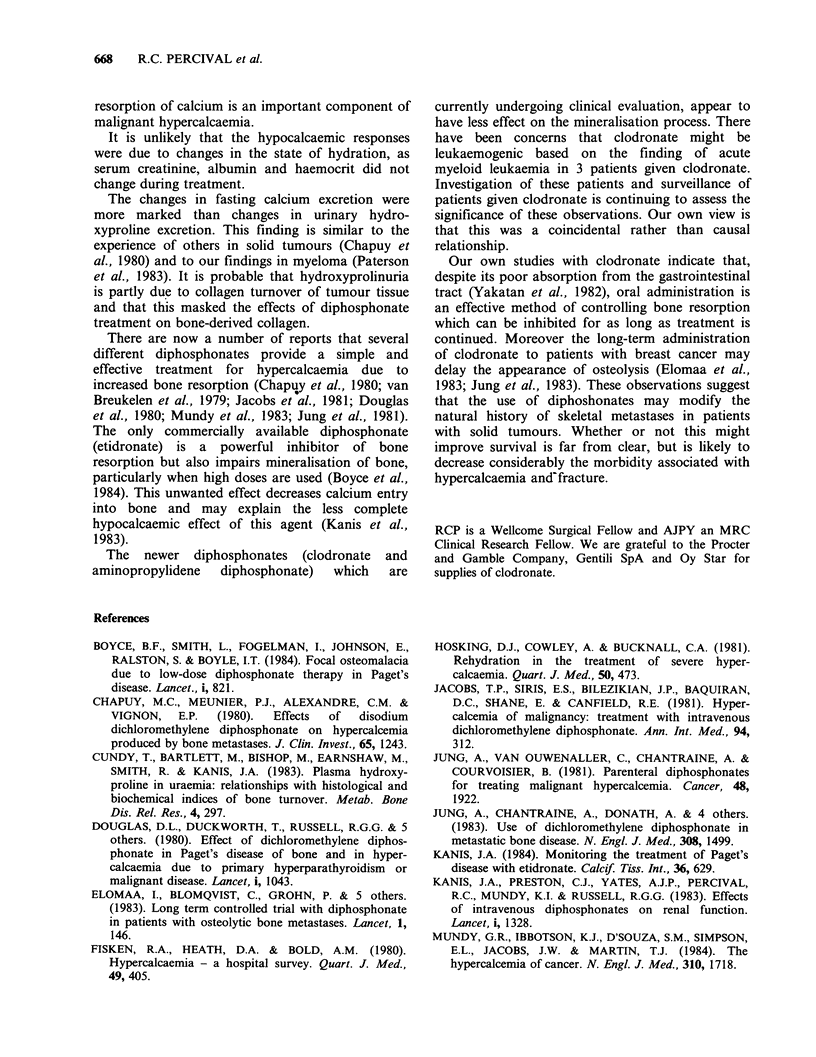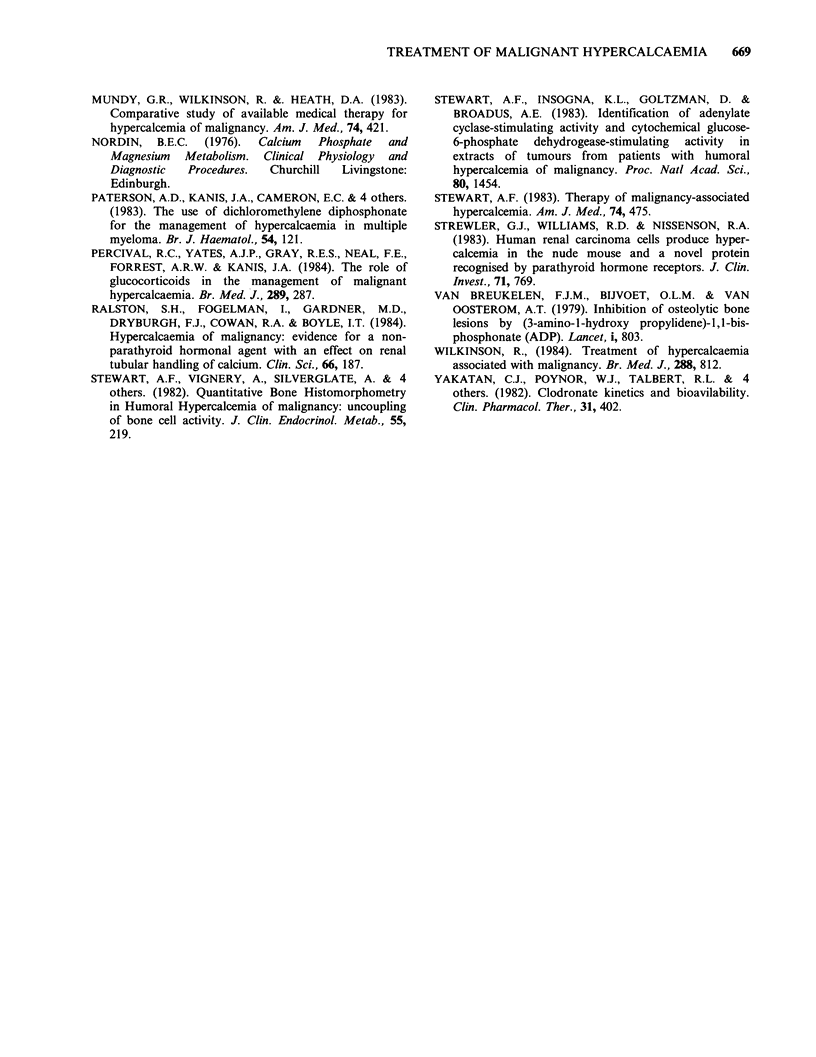# Treatment of malignant hypercalcaemia with clodronate.

**DOI:** 10.1038/bjc.1985.100

**Published:** 1985-05

**Authors:** R. C. Percival, A. D. Paterson, A. J. Yates, D. J. Beard, D. L. Douglas, F. E. Neal, R. G. Russell, J. A. Kanis

## Abstract

We have assessed the effects of clodronate (dichloromethylene diphosphonate; Cl2MDP 0.8-3.2g daily by mouth for up to 3 months) in 17 episodes of hypercalcaemia and osteolysis due to carcinoma. Clodronate reduced serum calcium in 14 episodes and bone resorption in all patients. These remained suppressed for the duration of treatment, but recurred promptly when treatment was stopped. Clodronate may be a useful measure for controlling hypercalcaemia and osteolysis in patients with carcinoma.


*Br. J. Cancer* (1985), **51**, 665–669

### Treatment of malignant hypercalcaemia with clodronate

R.C. Percival[1], A.D. Paterson[1], A.J.P. Yates[1], D.J. Beard[1], D.L. Douglas[1], F.E. Neal[2], R.G.G. Russell[1] & J.A. Kanis[1]

[1]*Department of Human Metabolism and Clinical Biochemistry, University of Sheffeld Medical School, Beech Hill Road, Sheffield S10 2RX and* [2]*Clinical Oncology and Radiotherapeutics Unit, Weston Park Hospital, Sheffield S10 2SJ, UK.*

**Summary** We have assessed the effects of clodronate (dichloromethylene diphosphonate; $Cl_2MDP$ 0.8–3.2 g daily by mouth for up to 3 months) in 17 episodes of hypercalcaemia and osteolysis due to carcinoma. Clodronate reduced serum calcium in 14 episodes and bone resorption in all patients. These remained suppressed for the duration of treatment, but recurred promptly when treatment was stopped. Clodronate may be a useful measure for controlling hypercalcaemia and osteolysis in patients with carcinoma.

Secondary carcinoma affecting the skeleton is the most common cause of hypercalcaemia in hospitalized patients (Fisken *et al.*, 1980), and usually indicates a poor prognosis. Prompt effective treatment may decrease morbidity allowing hospitalised patients to return home, and in some instances, enable them to tolerate additional treatment more readily. Our understanding of the pathogenesis of hypercalcaemia in carcinoma is incomplete. It is usually but not invariably associated with widespread skeletal metastases. The increased bone resorption may be accompanied by increased activity of osteoclasts but a direct effect of the tumour cells themselves on bone is also a possible mechanism (Stewart *et al.*, 1982). Rarely, hypercalcaemia is associated with increased bone resorption, but without obvious skeletal deposits (Mundy *et al.*, 1984) and is though to be mediated by humoral mechanisms not yet well characterised (Stewart *et al.*, 1983; Strewler *et al.*, 1983).

Adequate extracellular volume repletion is an important aspect of the treatment of hypercalcaemia which decreases renal tubular reabsorption of calcium and increases glomerular filtration and thus the filtered load of calcium (Hosking *et al.*, 1981). Agents which inhibit specifically osteoclast activity have also been used, including calcitonin, corticosteroids and mithramycin. However, the response to calcitonin is commonly variable and incomplete (Wilkinson, 1984) steroids are not always effective (Percival *et al.*, 1984; Mundy *et al.*, 1983) and mithramycin has toxic effects on bone marrow and liver (Stewart, 1983), particulary if used with other cytotoxic agents. More recently, the use of several diphosphonates, has given encouraging results in the treatment of hypercalcaemia (Jung *et al.*, 1981; Chapuy *et al.*, 1980; van Breukelen *et al.*, 1979). We have used clodronate (dichoromethylene diphosphonate) in patients with hypercalcaemia of various causes. Our results in myeloma have been reported elsewhere (Paterson *et al.*, 1983), and we report here our findings in patients with hypercalcaemia due to solid tumours.

### Patients and methods

Seventeen episodes of hypercalcaemia were studied in 15 patients (9 women and 6 men) with disseminated carcinoma before and after treatment with clodronate (Table I). Two patients (nos. 3 and 7) received a second course of clodronate which was separated by a treatment free interval of 5–6 weeks.

Patients were admitted to the study if their values for serum calcium were above normal (2.1–2.6 mmol $l^{-1}$) and either stable or rising in a 48 h control period despite adequate hydration. Six of the patients had received prednisolone (10–40 mg daily), but had failed to show any hypocalcaemic response despite treatment for 12 to 28 days. Where hypocalcaemic agents (including i.v. fluids or corticosteroids) were being administered in the period before treatment, these were continued in the same dose during the early period of treatment. All patients had scintigraphic or radiographic evidence of widespread skeletal metastases and one third had biochemical evidence for hepatic dysfunction.

Informed consent was obtained from all patients or from a relative where the patient was unfit to give consent. The study had the prior approval of the local Ethical Committee.

Correspondence: J.A. Kanis
Received 22 October 1984; and in revised form 21 January 1985.

© The Macmillan Press Ltd., 1985

**Table I** Details of patients studied

| Patient | Sex | Age | Primary carcinoma | Serum calcium ($mmol\,l^{-1}$) | Dose of clodronate ($g\,d^{-1}$) | Duration of treatment (weeks) | Concurrent therapy |
|---|---|---|---|---|---|---|---|
| 1 | F | 57 | Parathyroid | 3.79 | 3.2 | 10 | — |
| 2 | M | 55 | Bronchus | 2.91 | 3.2 | 0.5 | prednisolone |
| 3 | M | 72 | Larynx | 3.05 | 1.6 | 2 | prednisolone |
| | | | | 3.59 | 1.6 | 2 | |
| 4 | M | 62 | Bronchus | 3.69 | 3.2 | 12 | — |
| 5 | M | 50 | Adenocarcinoma (site unknown) | 2.94 | 1.6 | 6 | — |
| 6 | M | 48 | Hypernephroma | 3.27 | 3.2 | 10 | medroxyprogesterone |
| 7 | M | 72 | Prostate | 3.65 | 3.2 | 3 | — |
| | | | | 3.76 | 3.2 | 6 | — |
| 8 | F | 32 | Breast | 3.13 | 3.2 | 3 | prednisolone |
| 9 | F | 43 | Breast | 2.91 | 1.6 | 5 | prednisolone |
| 10 | F | 43 | Breast | 2.99 | 3.2 | 5 | — |
| 11 | F | 48 | Breast | 2.83 | 3.2 | 8 | prednisolone |
| 12 | F | 52 | Breast | 3.10 | 1.6 | 3 | — |
| 13 | F | 57 | Breast | 3.16 | 1.6 | 8 | — |
| 14 | F | 49 | Breast | 3.28 | 1.6 | 5 | prednisolone |
| 15 | F | 68 | Breast | 2.80 | 1.6 | 4 | — |

All patients were fully hydrated as judged by clinical criteria before the start of treatment with clodronate. Clodronate was given by mouth in a single daily dose of 0.8–3.2 g 2 h before breakfast. This range of dose was chosen because earlier studies had shown this to be effective in Paget's disease and hypercalcaemia due to myeloma (Douglas *et al.*, 1980; Paterson *et al.*, 1983). Patients were treated for periods ranging from 3 days to 3 months (Table I).

After an overnight fast, urine was collected during a 2 h period before breakfast. A venous blood sample was obtained during this period and the serum separated. Calcium, phosphate, creatinine and albumin were measured in serum by a Technicon SMAC Autoanalyser. Serum calcium was adjusted for variations in serum albumin by the addition or subtraction of $0.02\,mmol\,l^{-1}$ for each $g\,l^{-1}$ that albumin was below or above 42 g/l. Urinary calcium and hydroxyproline were expressed as ratios of urinary creatinine, which in the fasting state provided indices of net calcium release from bone and of bone resorption (Nordin, 1976; Cundy *et al.*, 1983).

The significance of changes in mean values was computed using Student's *t*-test for paired or non-paired observations as appropriate. Results are shown as means ($\pm$s.e.).

### Results

The administration of clodronate resulted in a progressive fall in serum calcium in 14 of the 17 episodes studied. The maximum effect on serum calcium was seen one week after starting treatment. Mean serum calcium fell from $3.23\pm0.08\,mmol\,l^{-1}$ to $2.85\pm0.09\,mmol\,l^{-1}$ at 1 week (Figure 1), and normal values for serum calcium were observed in 9 patients. In 14 studies (on 13 patients) treatment was continued for 3 to 10 weeks. In all but 4 patients a hypocalcaemic response was sustained for the duration of treatment though mean values rose slightly (Figure 1). Mean serum creatinine did not change throughout treatment ($130\pm20\,\mu mol\,l^{-1}$ before treatment and $137\pm19\,\mu mol\,l^{-1}$ at 1 week) and no changes in haematocrit or serum albumin were observed suggesting that changes in serum calcium could not be ascribed to changes in rehydration or to improved renal glomerular function. There was no difference in response in patients given concurrent corticosteroids. There was a consistent and significant fall in fasting urinary creatinine indicating a reduction in net bone loss, which persisted for the duration of treatment. Calciuria decreased to normal values in 75% of patients. Parallel but less marked decreases in urinary excretion of hydroxyproline were also observed. Both hypercalcaemia and a rise in calcium/creatinine ratio occured when treatment was stopped. Serum activity of alkaline phosphatase rose progressively throughout treatment and declined when treatment was stopped. This did not appear to be due to changes in hepatic function since no changes in the activity of hepatic transaminases was noted, and a marked increase in serum phosphatase activity was observed in 3

**Figure 1** Changes in mean serum calcium, serum alkaline phosphatase, fasting urinary calcium, and urinary hydroxyproline (SEM) in 17 episodes of malignant hypercalcaemia during treatment with clodronate, and in 11 episodes after stopping treatment. Asterisks denote significance of differences from values before or immediately after stopping treatment (*$P<0.05$; **$P<0.01$; ***$P<0.001$).

**Figure 2** Changes in serum calcium and fasting urinary calcium in a patient (no 12) who apparently failed to respond to treatment with clodronate. Note the fall in urinary calcium during treatment suggesting that clodronate inhibited bone resorption, but the lack of effect on serum calcium.

patients without other biochemical evidence of hepatic involvement.

No effects were noted on full blood counts. The only side effect noted was mild gastrointestinal upset in some patients.

Three patients failed to show a substantial fall in serum calcium. In two however, a marked fall in urinary calcium/creatinine was noted (eg Figure 2) suggesting that the reduction in bone resorption had been masked by a simultaneous rise in renal tubular reabsorption for calcium. The remaining patient failed to show any reduction in serum or urinary calcium possibly due to inadequate absorption of the drug.

### Discussion

These results indicate that clodronate given by mouth is an effective hypocalcaemic agent in patients with solid tumours. However, the magnitude of the hypocalcaemic response was less than in our own series of patients with hypercalcaemia due to myeloma treated identically with clodronate (Paterson *et al.*, 1983). Unfortunately, assays for the diphosphonates are not widely available nor easy to interpret (Kanis, 1985), so that it was not possible to document the bioavailibility of the drug. Despite this difficulty it is likely that clodronate decreased bone resorption to a similar extent in both myeloma and our patients with solid tumours. Thus net calcium release from bone, as judged by the calcium/creatinine ratio, was suppressed to a similar extent in myeloma and carcinoma, which suggests that the less complete response in patients with carcinoma was due to other mechanisms. Indeed, in 2 patients in whom plasma calcium did not change, there was evidence for effective suppression of excessive bone resorption. The lack of fall in serum calcium was probably due to to an increase in renal tubular reabsorption of calcium, and others (Ralston *et al.*, 1984) have suggested that increased renal tubular resorption of calcium is an important component of malignant hypercalcaemia.

It is unlikely that the hypocalcaemic responses were due to changes in the state of hydration, as serum creatinine, albumin and haemocrit did not change during treatment.

The changes in fasting calcium excretion were more marked than changes in urinary hydroxyproline excretion. This finding is similar to the experience of others in solid tumours (Chapuy *et al.*, 1980) and to our findings in myeloma (Paterson *et al.*, 1983). It is probable that hydroxyprolinuria is partly due to collagen turnover of tumour tissue and that this masked the effects of diphosphonate treatment on bone-derived collagen.

There are now a number of reports that several different diphosphonates provide a simple and effective treatment for hypercalcaemia due to increased bone resorption (Chapuy *et al.*, 1980; van Breukelen *et al.*, 1979; Jacobs *et al.*, 1981; Douglas *et al.*, 1980; Mundy *et al.*, 1983; Jung *et al.*, 1981). The only commercially available diphosphonate (etidronate) is a powerful inhibitor of bone resorption but also impairs mineralisation of bone, particularly when high doses are used (Boyce *et al.*, 1984). This unwanted effect decreases calcium entry into bone and may explain the less complete hypocalcaemic effect of this agent (Kanis *et al.*, 1983).

The newer diphosphonates (clodronate and aminopropylidene diphosphonate) which are currently undergoing clinical evaluation, appear to have less effect on the mineralisation process. There have been concerns that clodronate might be leukaemogenic based on the finding of acute myeloid leukaemia in 3 patients given clodronate. Investigation of these patients and surveillance of patients given clodronate is continuing to assess the significance of these observations. Our own view is that this was a coincidental rather than causal relationship.

Our own studies with clodronate indicate that, despite its poor absorption from the gastrointestinal tract (Yakatan *et al.*, 1982), oral administration is an effective method of controlling bone resorption which can be inhibited for as long as treatment is continued. Moreover the long-term administration of clodronate to patients with breast cancer may delay the appearance of osteolysis (Elomaa *et al.*, 1983; Jung *et al.*, 1983). These observations suggest that the use of diphoshonates may modify the natural history of skeletal metastases in patients with solid tumours. Whether or not this might improve survival is far from clear, but is likely to decrease considerably the morbidity associated with hypercalcaemia and fracture.

RCP is a Wellcome Surgical Fellow and AJPY an MRC Clinical Research Fellow. We are grateful to the Procter and Gamble Company, Gentili SpA and Oy Star for supplies of clodronate.

### References

BOYCE, B.F., SMITH, L., FOGELMAN, I., JOHNSON, E., RALSTON, S. & BOYLE, I.T. (1984). Focal osteomalacia due to low-dose diphosphonate therapy in Paget's disease. *Lancet.*, **i**, 821.

CHAPUY, M.C., MEUNIER, P.J., ALEXANDRE, C.M. & VIGNON, E.P. (1980). Effects of disodium dichloromethylene diphosphonate on hypercalcemia produced by bone metastases. *J. Clin. Invest.*, **65**, 1243.

CUNDY, T., BARTLETT, M., BISHOP, M., EARNSHAW, M., SMITH, R. & KANIS, J.A. (1983). Plasma hydroxyproline in uraemia: relationships with histological and biochemical indices of bone turnover. *Metab. Bone Dis. Rel. Res.*, **4**, 297.

DOUGLAS, D.L., DUCKWORTH, T., RUSSELL, R.G.G. & 5 others. (1980). Effect of dichloromethylene diphosphonate in Paget's disease of bone and in hypercalcaemia due to primary hyperparathyroidism or malignant disease. *Lancet*, **i**, 1043.

ELOMAA, I., BLOMQVIST, C., GROHN, P. & 5 others. (1983). Long term controlled trial with diphosphonate in patients with osteolytic bone metastases. *Lancet*, **1**, 146.

FISKEN, R.A., HEATH, D.A. & BOLD, A.M. (1980). Hypercalcaemia – a hospital survey. *Quart. J. Med.*, **49**, 405.

HOSKING, D.J., COWLEY, A. & BUCKNALL, C.A. (1981). Rehydration in the treatment of severe hypercalcaemia. *Quart. J. Med.*, **50**, 473.

JACOBS, T.P., SIRIS, E.S., BILEZIKIAN, J.P., BAQUIRAN, D.C., SHANE, E. & CANFIELD, R.E. (1981). Hypercalcemia of malignancy: treatment with intravenous dichloromethylene diphosphonate. *Ann. Int. Med.*, **94**, 312.

JUNG, A., VAN OUWENALLER, C., CHANTRAINE, A. & COURVOISIER, B. (1981). Parenteral diphosphonates for treating malignant hypercalcemia. *Cancer*, **48**, 1922.

JUNG, A., CHANTRAINE, A., DONATH, A. & 4 others. (1983). Use of dichloromethylene diphosphonate in metastatic bone disease. *N. Engl. J. Med.*, **308**, 1499.

KANIS, J.A. (1984). Monitoring the treatment of Paget's disease with etidronate. *Calcif. Tiss. Int.*, **36**, 629.

KANIS, J.A., PRESTON, C.J., YATES, A.J.P., PERCIVAL, R.C., MUNDY, K.I. & RUSSELL, R.G.G. (1983). Effects of intravenous diphosphonates on renal function. *Lancet*, **i**, 1328.

MUNDY, G.R., IBBOTSON, K.J., D'SOUZA, S.M., SIMPSON, E.L., JACOBS, J.W. & MARTIN, T.J. (1984). The hypercalcemia of cancer. *N. Engl. J. Med.*, **310**, 1718.

MUNDY, G.R., WILKINSON, R. &. HEATH, D.A. (1983). Comparative study of available medical therapy for hypercalcemia of malignancy. *Am. J. Med.*, **74**, 421.

NORDIN, B.E.C. (1976). *Calcium Phosphate and Magnesium Metabolism. Clinical Physiology and Diagnostic Procedures.* Churchill Livingstone: Edinburgh.

PATERSON, A.D., KANIS, J.A., CAMERON, E.C. & 4 others. (1983). The use of dichloromethylene diphosphonate for the management of hypercalcaemia in multiple myeloma. *Br. J. Haematol.*, **54**, 121.

PERCIVAL, R.C., YATES, A.J.P., GRAY, R.E.S., NEAL, F.E., FORREST, A.R.W. & KANIS, J.A. (1984). The role of glucocorticoids in the management of malignant hypercalcaemia. *Br. Med. J.*, **289**, 287.

RALSTON, S.H., FOGELMAN, I., GARDNER, M.D., DRYBURGH, F.J., COWAN, R.A. & BOYLE, I.T. (1984). Hypercalcaemia of malignancy: evidence for a non-parathyroid hormonal agent with an effect on renal tubular handling of calcium. *Clin. Sci.*, **66**, 187.

STEWART, A.F., VIGNERY, A., SILVERGLATE, A. & 4 others. (1982). Quantitative Bone Histomorphometry in Humoral Hypercalcemia of malignancy: uncoupling of bone cell activity. *J. Clin. Endocrinol. Metab.*, **55**, 219.

STEWART, A.F., INSOGNA, K.L., GOLTZMAN, D. & BROADUS, A.E. (1983). Identification of adenylate cyclase-stimulating activity and cytochemical glucose-6-phosphate dehydrogease-stimulating activity in extracts of tumours from patients with humoral hypercalcemia of malignancy. *Proc. Natl Acad. Sci.*, **80**, 1454.

STEWART, A.F. (1983). Therapy of malignancy-associated hypercalcemia. *Am. J. Med.*, **74**, 475.

STREWLER, G.J., WILLIAMS, R.D. & NISSENSON, R.A. (1983). Human renal carcinoma cells produce hypercalcemia in the nude mouse and a novel protein recognised by parathyroid hormone receptors. *J. Clin. Invest.*, **71**, 769.

VAN BREUKELEN, F.J.M., BIJVOET, O.L.M. & VAN OOSTEROM, A.T. (1979). Inhibition of osteolytic bone lesions by (3-amino-1-hydroxy propylidene)-1,1-bis-phosphonate (ADP). *Lancet*, **i**, 803.

WILKINSON, R., (1984). Treatment of hypercalcaemia associated with malignancy. *Br. Med. J.*, **288**, 812.

YAKATAN, C.J., POYNOR, W.J., TALBERT, R.L. & 4 others. (1982). Clodronate kinetics and bioavilability. *Clin. Pharmacol. Ther.*, **31**, 402.